# iPSCs in Modeling and Therapy of Osteoarthritis

**DOI:** 10.3390/biomedicines9020186

**Published:** 2021-02-12

**Authors:** Maria Csobonyeiova, Stefan Polak, Andreas Nicodemou, Radoslav Zamborsky, Lubos Danisovic

**Affiliations:** 1 Institute of Histology and Embryology, Faculty of Medicine, Comenius University, Sasinkova 4, 811 08 Bratislava, Slovakia; maria.csobonyeiova@fmed.uniba.sk (M.C.); stefan.polak@fmed.uniba.sk (S.P.); 2 Institute of Medical Biology, Genetics and Clinical Genetics, Faculty of Medicine, Comenius University, Sasinkova 4, 811 08 Bratislava, Slovakia; andreas.nicodemou@fmed.uniba.sk; 3 National Institute of Children’s Diseases, Department of Orthopedics, Faculty of Medicine, Comenius University, Limbova 1, 833 40 Bratislava, Slovakia; radozamborsky@gmail.com; 4 Regenmed Ltd., Medena 29, 811 01 Bratislava, Slovakia

**Keywords:** osteoarthritis, iPSCs, articular cartilage, stem cell-based therapy, disease modeling

## Abstract

Osteoarthritis (OA) belongs to chronic degenerative disorders and is often a leading cause of disability in elderly patients. Typically, OA is manifested by articular cartilage erosion, pain, stiffness, and crepitus. Currently, the treatment options are limited, relying mostly on pharmacological therapy, which is often related to numerous complications. The proper management of the disease is challenging because of the poor regenerative capacity of articular cartilage. During the last decade, cell-based approaches such as implantation of autologous chondrocytes or mesenchymal stem cells (MSCs) have shown promising results. However, the mentioned techniques face their hurdles (cell harvesting, low proliferation capacity). The invention of induced pluripotent stem cells (iPSCs) has created new opportunities to increase the efficacy of the cartilage healing process. iPSCs may represent an unlimited source of chondrocytes derived from a patient’s somatic cells, circumventing ethical and immunological issues. Aside from the regenerative potential of iPSCs, stem cell-derived cartilage tissue models could be a useful tool for studying the pathological process of OA. In our recent article, we reviewed the progress in chondrocyte differentiation techniques, disease modeling, and the current status of iPSC-based regenerative therapy of OA.

## 1. Introduction

OA is the most frequent chronic joint disease present in the majority of individuals over the age of 65. It typically affects the knees, hands, hips, and spine, causing degenerative lesions of the cartilage, subchondral bone, and other joint tissues leading to impaired mobility in older people. The most common symptoms include joint swelling, joint deformities, chronic pain, stiffness, and tenderness. Typical for more progressed stages is the degeneration of ligaments and menisci together with hypertrophy of the joint capsule. To understand OA’s pathology, it is essential to mention that diarthrodial joins are composed of articular cartilage (AC). The AC lacks blood vessels, nerves, and it is hypocellular; therefore, its nutrition relies on synovial fluid diffusion powered by joint movements. The extracellular matrix produced by chondrocytes is very dense, so in the case of cartilage injury, the progenitor cells cannot penetrate within. Due to this specific AC feature, the extracellular matrix’s turnover is minimal, hindering cartilage regeneration. The development of OA is associated with several risk factors disturbing the articular chondrocyte homeostasis including genetic predisposition, aging, obesity, inflammation, repetitive stress injury, physical inactivity, and chronic stress [1,2,3].

Some recent studies have reported that several cytokines and growth factors are involved in the process of articular cartilage destruction. During the initial stages of OA, the chondrocytes release oxygen free radicals, which are responsible for progressive cartilage damage. Furthermore, the role of fibronectin in matrix degradation was also reported [4,5]. Many inflammatory cytokines such as tumor necrosis factor-alpha (TNF-α), interleukin (IL)-6, and IL-1 together with degenerative enzymes including metalloproteinase, aggrecanases, and collagenase are responsible for significant proteoglycan and collagen breakdown. Subsequently, the chondrocytes undergo apoptosis, and the cartilage is ultimately lost. However, the precise molecular mechanism of the pathological process lying behind OA remains obscure [6]. 

There are currently no effective therapies that can prevent OA development or reverse the progression of the disease. Traditional treatment relies only on pharmacological intervention, primarily used for pain management and reducing stiffness to improve quality of life. For patients in more advanced stages, there is a possibility for total joint replacement surgery; therefore, there has been a need for less invasive therapeutic options such as the use of stem cells with a combination of growth factors and scaffolds. Nowadays, the main focus of stem cell-based therapy of OA represents the autologous chondrocytes and mesenchymal stem cell transplantation, which can slow down the progression of the disease and delay surgery, although the use of autologous chondrocytes is often related to donor site morbidity including hypertrophy and graft failure. Regarding the MSCs, their heterogeneity and limited proliferation capacity decrease their regenerative efficacy [7,8]. 

Using induced pluripotent stem cells (iPSCs) may overcome many of the mentioned issues. The invention of iPSCs by a team of Nobel Prize winner, Shinya Yamanaka, has rapidly revolutionized the field of regenerative medicine and tissue engineering [9]. The iPSCs were initially generated from somatic cells by the viral transfection of key reprogramming factors (Oct4, Sox2, Klf4, c-myc) into the donor cells. However, the viral methods (retroviruses, lentiviruses) were accompanied by serious risks of insertional mutagenesis and reactivation of transgenes caused by the integration of the viral genome, which may be responsible for tumor formation [10]. To overcome this issue, safer integration-free techniques such as plasmid vectors [11], episomal plasmid vectors [12], piggyBac transposons [13], Sendai virus [14], microRNAs [15], synthetic mRNA [16], protein-based methods [17], and small molecules [18] have been developed. Important to say is that all available methods differ in efficiency, quality, and costs.

The iPSCs are similar to the ESCs in their pluripotency, cell morphology, gene expression, and proliferation capability; however, the iPSCs are derived from patient’s somatic cells circumventing the ethical issues related to the use of ESCs. The unique feature of iPSCs is their patient-specificity, minimizing the autoimmune response, making them an almost ideal cell source for cell-based therapy.

## 2. Induced Pluripotent Stem Cell (iPSC)-Based Osteoarthritis Modeling

The iPSCs represent a promising tool to investigate the pathology behind the disease mechanism and evaluate the pros and cons of their possible clinical application on iPSC-based models for osteodegenerative diseases such as OA (Table 1). The potential of iPSCs to differentiate into the chondrocytes and their application in disease modeling has been successfully demonstrated in several studies [19,20,21]. For instance, Diekman et al. [22] established an in vitro cartilage defect model to investigate the regenerative potential of iPSC-derived chondrocytes. The chondrogenetically differentiated and purified iPSCs seeded in agarose started to produce a cartilage matrix one week after seeding, thus demonstrating their reparative ability. Xu et al. [21], derived iPSCs from patients suffering from familial osteochondritis dissecans, an inherited disease characterized by early onset of severe OA. First, the patient-specific iPSCs were injected subcutaneously into immunodeficient mice. In the second step, the teratoma tissue was harvested after 2–3 months, and the cartilage tissue was identified by Safranin-O staining. The iPSC-derived OA cartilage model displayed typical OA pathology such as poor matrix formation and accumulation of aggrecan within the endoplasmic reticulum of chondrocytes, but no presence of aggrecan in extracellular mass, making the cartilage more vulnerable to structural damage. Despite the successful cartilage formation from iPSC-derived teratoma tissue, this method is not suitable for replacement therapies for OA patients due to transplant animal growth as well as the time- and cost-consuming differentiation process [23]. Recently, the research group of Rim et al. [24] proved the therapeutic effect of iPSC-derived chondrocytes delivered only by single intra-articular injection into the rat model with the osteochondral defect. Eight weeks post-transplantation, the high recovery capacity of injected iPSC-derived chondrocytes, which formed lacunae in vivo, were detected. Further analyses showed positivity for filaggrin and CD55 as well as high expression of collagen type I. This novel non-invasive cell implantation approach represents a promising alternative to replace surgical intervention in cartilage regeneration therapy. 

The effectiveness of OA therapeutics on the in vitro iPSC-derived OA cartilage model was investigated by Willard et al. [25]. Before drug screening, the iPSCs cartilage models were treated with the cytokine interleukin-1α for 21 days to induce characteristic OA pathological phenotypes such as the loss of tissue mechanical properties and elevated level of inflammatory mediators. For high-throughput screening, the authors selected five disease-modifying drug candidates including IL-4, tissue inhibitor of metalloproteinase-3, the COX-2 inhibitor NS-398, NF-κB inhibitor SC-514, and MMP inhibitor GM-6001. According to media analyses, all the examined compounds displayed some protective features. However, SC-514 significantly increased a vast range of protective pathways such as a reduction of GAG loss as well as a decrease in MMP production, NO production, and PEG2 production. Based on published results, the authors concluded that such an iPSC-derived OA model could represent a novel platform for robust screening of potential new therapeutic agents for OA patients.

An innovative OA model derived from iPSCs without the involvement of animals was developed by Lin et al. [26]. The authors created microphysiological osteochondral tissue chips through the differentiation of iPSCs into mesenchymal progenitor cells (iMPCs), which were encapsulated within the gelatin scaffolds. The cultivation was performed in a dual-flow bioreactor with both chondrogenic and osteogenic media. After 28 days of cultivation, the tissue chips were successfully formed. For the OA pathology induction in the cartilage side of the chip, interleukin-1β was added to the cultivation media, resulting in a decrease of COL2 and ACAN expression and enhanced tissue degeneration. Furthermore, the authors decided to confirm the efficacy of the chip OA models by testing the non-steroidal anti-inflammatory drug Celecoxib, a COX-2 inhibitor, which is often prescribed to OA patients. After one-week of Celecoxib treatment, results showed notable suppression of inflammatory factors, IL-1β, IL-6, and COX2, together with a partial retrieve of COL2 and ACAN levels, showing its osteoprotective abilities. Taken together, organ-on-a-chip technology has attracted a lot of attention in the field of disease modeling and drug screening in recent years because of its advantage in studying different tissue interactions on cellular and intracellular levels.

Since OA is a systemic pathologic condition and a complex disease affected by several factors (genetic and environmental), it is challenging to generate an in vitro model [27]. The pathology of OA does not involve only the articular cartilage defects, but the whole joint including perichondrium, menisci, bone, synovial membrane, ligaments, and muscle. Therefore, it is not entirely sufficient to generate only in vitro cartilage models. On the other hand, such models can be very valuable in studying the early development of OA.

## 3. Differentiation of iPSCs into Chondrocytes

The initial attempts to differentiate chondrocytes from human OA-derived iPSCs were published just a few years ago (Table 2). The basic principle of differentiation protocols is to convert cell fate toward chondrogenic lineage; therefore, the culture medium is usually supplemented with growth factors such as TGF-β, BMP, WNT3A, and FGF-2. A short time ago, it was found that the paracrine factors such as Ihh and Runx also influence chondrogenic differentiation of iPSCs [28,29,30]. There are currently four main chondrogenic differentiation approaches including (a) the generation of MSC-like iPSCs and their subsequent differentiation into chondrocytes [31,32]; (b) the co-culture of iPSCs-derived MSCs with primary chondrocytes [33]; (c) through the formation of embryoid bodies (EBs) [19,24,34,35]; and (d) culturing of iPSCs in a series of media architected to mimic normal developmental pathways [20,22]. iPSC formation through EBs together with their subsequent co-cultivation with chondrocytes was performed by Wei et al. [35]. First, the chondrocytes obtained from OA patients were reprogrammed to OA-iPSCs by lentiviral induction. The second step involved the formation of EBs, followed by 14 days of cultivation in the chondrogenic medium. After that, iPSCs were transfected with lentivirus carrying TGF-1β and seeded onto the alginate matrix coated dishes. After another 14 days of cultivation, the TGF-1β/iPSCs were injected subcutaneously into the dorsal region of mice. At the sixth week post-transplantation, the grafts were analyzed and showed the presence of ectopic cartilage tissue formation. 

**Figure 1:**
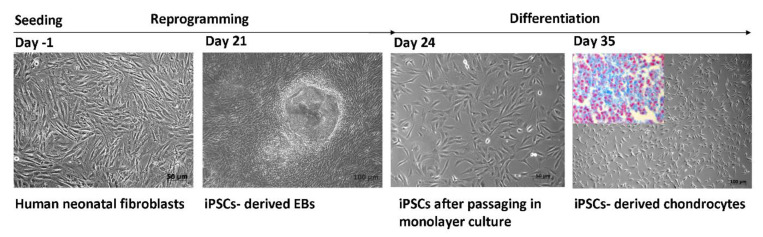
The generation of induced pluripotent stem cell (iPSC)-derived chondrocytes through the embryoid bodies (EBs) method.

Our research group also performed the EBs method for chondrocyte differentiation [40]. The reprogramming of long-term preserved human neonatal fibroblasts into iPSCs was done by a transgene-free combination of episomal vectors transfected by the Lipofectamine 3000 transfection system. Within 14 days of cultivation, the iPSC colonies underwent chondrogenic differentiation in vitro, which was impelled in pellet cultures using chondrogenic differentiation media (StemMACS ChondroDiff Media, Miltenyi Biotec, Bergisch Gladbach, Germany). The quantitative real-time PCR analyses, accomplished after three weeks of cultivation, showed significantly higher expression of Col2a1 compared to the control (Figure 1). 

A comparable method was published Li et al. [41], however, with a different primary cell source. This study aimed to reprogram peripheral blood cells (PBCs) into iPSCs and induce chondrogenic differentiation to prove the capability of PBCs as seed cells to replace the use of fibroblasts and differentiate into chondrocytes. The reason for replacement was to circumvent some issues related to the often painful biopsy of tissue and in vitro expansion of fibroblasts. The PBCs were reprogrammed to iPSCs via transgene-free episomal vectors and spontaneously differentiated into EBs using EB formation medium and basal culture medium. Within ten days, the fibroblast-like cells were observed, and undifferentiated cells were excluded. In order to accomplish chondrogenic differentiation, the fibroblast-like cells were cultured in a chondrogenic medium via 3D pellet culture, supplemented with TGF-β1 and dexamethasone for 21 days. To avoid using the animal components of FBS, the insulin-transferrin-selenium supplement (10% concentration) was applied, contributing to chondrocyte differentiation. The final populations of iPSC-derived chondrogenic cells were positive to Alcian blue and toluidine blue and expressed *Col2, Col10, SOX9,* and *Aggrecan,* suggesting successful differentiation. Therefore, the PBCs represent an appropriate alternative to fibroblasts in terms of reprogramming and patient-specific chondrocyte generation, whereas only 2 mL of blood could be sufficient to generate iPSC-derived chondrocytes to repair cartilage defect. To another primary cell candidate suitable for chondrogenic differentiation belong the cord blood mononuclear cells (CBMCs). According to the research of Nam et al. [42], the CBMCs-iPSCs may offer similar cell properties for reprogramming and chondrogenic differentiation as PBCs and fibroblasts. In his work, the EBs derived from the CBMCs-iPSCs were similar to those in the study of Li et al. (2017) [41], cultured in chondrogenic medium with pellet culture supplemented with TGF-β3. After 30 days, the differentiated chondrogenic pellets were analyzed for the presence of cartilage-specific markers (ACAN, COMP, Col2A1, and SOX9) with positive results. The production of the extracellular matrix was also confirmed by staining methods. 

The generation of iPSC-derived chondrocytes through the EBs method was improved by Zhu et al. [19]. In their simple 3-step protocol, the dermal fibroblasts from OA patients were reprogramed into iPSCs, and afterward, the iPSCs colonies were treated toward the EBs with 0.5 mg/mL dispase for five days. Subsequently, the EB medium was changed to chondrogenic media, and cultivation continued for two days. The last step involved the cultivation of EBs in gelatin-coated dishes with chondrogenic media for another one or two weeks, resulting in several populations of cells with chondrocyte-like morphology. The following analyses such as toluidine blue and collagen type II positivity, and expression of Col2A1, ACAN, and SOX9 revealed the chondrogenic capacity of differentiated iPSCs. To evaluate the in vivo regenerative potential of iPSC-derived chondrocytes, researchers injected differentiated cells into the MIA-induced OA rat model. After 15 weeks, the histological examination was conducted and showed increased production of aggrecan and collagen. Rats significantly improved their movement abilities; however, persistent dyskinesia indicated incomplete joint recovery.

It is well known that micro-RNAs play a crucial role, particularly in the chondrogenesis of stem cells [43]. More recently, Mahboudi et al. [44] established a novel differentiation protocol in which the iPSC-derived EBs were infected with the miR-140 recombinant lentiviral vector and cultured in a medium supplemented with BMP2 and TGF-β3. The authors based the present protocol on their previous work focused on the chondrogenic potential of micro-RNA overexpression in MSCs [36]. Furthermore, the chondrogenic differentiation of iPSCs was enhanced by a high cell-density culture system. Real-time RT-PCR analyses performed on days 7, 14, and 21 revealed upregulation of Col2, ColX, and aggrecan genes; on the other hand, the expression of Col1 was downregulated. Formation of cartilage with typical components of the extracellular matrix was proved by positive Alcian blue stain. Considering the mentioned results, the combination of miR-140 overexpression and TGF-β3 seems to be a compelling chondrogenic inducer with higher efficacy over the use of TGF-β3 alone.

Nejadnik et al. [32] published a highly efficient differentiation protocol without the need for the formation of EBs, which is usually responsible for the heterogeneous cell population. The authors developed a differentiation method based on the direct induction of iPSCs into iPSC-MSCs, followed by chondrogenic differentiation. The iPSCs were cultured in hMSC medium. On day 21, iPSC-MSCs exhibited spindle-shaped morphology and were positive for MSCs surface markers CD105, CD73, and CD90. Subsequently, the medium was changed to a serum-free chondrogenic differentiation medium supplemented with TGF-β3. On day 14, the cartilage markers Col2A1, Col9A1, Col11A1, SOX9, and ACAN were expressed. After seven more days of cultivation, the histological analyses showed positivity for the Alcian blue stain, demonstrating proteoglycan production. Cartilage matrix formation was proven by collagen type II positivity. After that, the iPSC-derived chondrogenic pellets were implanted into the osteochondral defect of the distal femur of nude athymic rats. After six weeks, successful remodeling of the defect and active production of the chondrogenic matrix was detected.

All mentioned techniques have been quite successful; however, they possess several limitations resulting in heterogeneous cell populations with low potential to form healthy cartilage. The more promising method, resulting in the generation of pure cartilage formation in vivo, was published by Yamashita et al. [18]. Researchers generated homogenous cartilaginous particles from human iPSC-derived mesodermal cells using scaffoldless suspension culture supplemented with BMP2, TGF-β1, and GDF5. The iPSC-derived cartilaginous particles were transplanted subcutaneously into severe combined immunodeficiency (SCID) mice and joint defects of immunodeficient rats and mini-pigs. They performed histological analyses in SCID mice 12 weeks after transplantation, indicating cartilage tissue formation with high intensity to safranin O staining and positive collagen type II expression. Additionally, no tumor formation and ectopic tissue were detected. Similar to the transplantation of cartilaginous particles in the joint defect of rats, the implanted tissue exhibited a strong expression of collagen type II, and it did not show any tumor or ectopic tissue formation within 28 days post-transplantation. The iPSC-derived cartilaginous particles transplanted into knee joint defects of mini-pigs filled the defect with cartilaginous tissue and survived up to one month. Taken together, all experiments showed successful neocartilage formation with the potential to integrate into native cartilage.

Another interesting study was published by Saito et al. [36]. The authors applied a feeder-free chondrogenic differentiation protocol based on the use of seven cytokines, which was previously established for ESCs by Oldershaw et al. [45]. Initially, the cells were pre-treated with a medium supplemented with FGF2. After one week, the iPSCs were cultured with chondrogenic differentiation medium including activin-A, WNT3A, Follistatin, BMP4, GDF5, and FGF2 neurotrophin 4. The successful differentiation of iPSCs to chondrocytes was proven by the expression of chondrocyte marker genes–SOX9, SOX6, Col2A1, and ACAN. The iPSC-derived chondrocytes formed cartilage disks within one week in a 3D culture system using a cylindrical mold. For the transplantation, a 1 mm cylindrical cartilage disk was cut off. Eight weeks after transplantation into the cartilage defect of NOD/SCID mice, a large amount of hyaline cartilage formation and regeneration with no abnormal tissue was observed. However, one large tumor had developed in a single mouse at 16 weeks. The authors suggested that the tumor was probably formed from immature iPSCs3; thus, the major attention must be focused on the purification of fully differentiated iPSC-derived chondrocytes before transplantation.The efficacy of four chondrogenic differentiation methods was examined by the group of Suchorska et al. [46]. Chosen methods included: (a) iPSCs cultured in monolayer with defined GFs; (b) iPSC- derived EBs in chondrogenic medium with TGF-β3; (c) iPSC- derived EBs in chondrogenic medium co-cultured with human chondrocytes and; (d) iPSC- derived EBs in chondrogenic medium co-cultured with human chondrocytes and supplemented with TGF-β3. According to RT-PCR, immunofluorescence, and flow cytometry analyses among the methods mentioned above are the most effective and less time-consuming in both the direct monolayer culture method with defined GFs and EBs cultured in medium conditioned with human chondrocytes. Moreover, the direct method’s advantage is that there is no need for an additional step, such as EBs formations. 

Yet, there is no definitive uniform differentiation method for the most effective iPSC- derived chondrocytes generation, which should be beneficial for articular cartilage regeneration. Therefore, more in vitro studies incorporating tissue engineering techniques are required to find the most suitable chondrogenic differentiation protocol producing functional and stable extracellular matrix secreting chondrocytes mimicking the native articular chondrocytes with the high-throughput outcome. 

## 4. Alternative Approaches in iPSC-Based Cartilage Reconstruction

During the past few years, tissue engineering methods such as 3D bioprinting have shown favorable results in the cartilage formation process. 3D bioprinting allows the distribution of different cell types on supporting biomaterials in a way resembling native tissue microarchitecture. The research group of Nguyen et al. [37] bioprinted iPSCs using a nanofibrillated cellulose + alginate (NFC/A) composite bio-ink co-printed with irradiated human chondrocytes. Within five weeks, the hyaline-like cartilaginous tissue with an increased number of chondrocytes was observed in bioprinted constructs. These results suggest that 3D bioprinting of iPSCs and their direct differentiation into chondrocytes using the most appropriate bio-ink could represent a new regeneration therapy for injured cartilage.

Most recently, the research has focused on finding suitable biomaterials to facilitate cartilage repair in combination with iPSCs, thus replacing time-consuming multiple differentiation-step protocols. Until today, there have been only a few published attempts at combing biomaterials with iPSCs to promote chondrogenic differentiation. For instance, Hontani et al. [38] came up with a novel chondrogenic differentiation method based on a combination of 3D cultivation in ultra-purified alginate gel as a scaffold and step-wise differentiation of iPSCs into chondrocytes via the MSC-like cell stage. iPSC-MSCs in alginate gel were supplemented with MSC induction medium. The initial medium was switched to the chondrogenic medium one day after the appearance of alginate beads. The following day, the human recombinant BMP-2 was added to the medium. The expression of typical chondrogenic markers in iPSC-MSCs such as SOX9, Col2A1, and aggrecan notably increased over time of cultivation. Chondrogenic differentiation in vivo was performed on nude mouse models through subcutaneous transplantation of iPSC-MSCs embedded in alginate gel beads into the dorsal flank. After several days, the histologic examination of grafts showed positivity for Alcian blue; however, very low intensity for type II collagen staining. Moreover, the expression of SOX9 was not elevated. The authors hypothesized that the cause of insufficient features could be subcutaneous graft transplantation with possible host cell contamination. This differentiation protocol shows clear improvement in the specificity of the chondrogenic differentiation of iPSCs compared to other conventional methods. 

Hu et al. [39] engineered bio-active lithium-containing bioceramics (L2C4S4) for direct chondrogenic differentiation of iPSCs. Results showed that in comparison with traditional chondrocyte-induction medium, the iPSCs cultivated in L2C4S4 biomaterial extracts expressed accelerated chondrogenic differentiation within 14 days and prevented the hypertrophy of differentiated chondrocytes. The mentioned study offers a new strategy for auricular cartilage regeneration comparable to traditional methods.

Biomechanical properties belong to the most important characteristics of tissue engineered cartilage, which influence its utilization in the regeneration of damaged cartilage. Middendorf et al. [47] prepared cartilage from chondrocytes differentiated from iPSCs. They evaluated the mechanics (compression, friction, and shear modulus) of their constructs in comparison with clinically relevant chondrocyte seeded constructs. Based on their results, it can be emphasized that artificial cartilage from iPSCs derived chondrocytes have similar or superior biomechanical characteristics and after performing further studies dealing with the optimization of culture conditions (e.g., application of variety culture media and growth factors, dynamic cultivation in bioreactors, etc.) may accelerate their translation into human medicine. 

## 5. Conclusions and Future Perspectives

Progress in iPSC generation methods has rapidly enhanced the field of stem cell-based therapies in a number of diseases. Regarding the OA, most clinical efforts have focused on restoration/regeneration of damaged articular cartilage using tissue engineering techniques together with cell-based approaches. Until today, several multi-step chondrocyte differentiation methods have been developed, and their quality has been improved in recent years. For instance, one of the auspicious approaches represents a new technique based on high-throughput iPSC-derived tissue chip technology enabling precise creation of microphysiological OA models of the human articular joint with high therapeutic effect on drug screening. 

Nevertheless, a complete reprogramming and differentiation process resulting in a functionally stable population of iPSC-derived chondrocytes is still quite time- and cost-consuming. The differentiation time ranges from 21 days up to almost 50 days; however, new differentiation techniques using bioceramics have significantly accelerated differentiation, reducing the time to 14 days. 

Moreover, for the future applications of iPSC-derived chondrocytes in OA replacement therapy, there is a need to solve safety issues concerning the risk of teratoma formation and immune response. iPSCs themselves possess genetic variations hindering their use in novel therapies; despite that, not all cancer mutations lead directly to tumorigenesis. Therefore, more precise validation experiments could exclude these possible harmful mutations. Nowadays, the approach focus on increasing the safety of the reprogramming process represents the use of a suitable cell source and the application of non-viral transfection methods, together with the replacement of potent oncogenes with small molecules and optimal passage number. It is well-known that likewise, the primary cell source can influence reprogramming and differentiation success. In the case of chondrogenic differentiation, several studies have examined different primary types of cells, among which the PBCs and CBMCs represent at least a similar candidate to widely using fibroblasts. Moreover, it is much easier and less invasive to obtain PBCs than fibroblasts. Advantages of CBMCs rely on the cell-banking system and stored HLA-typing information. Nowadays, it is well-known that the HLA mismatch is one of the major impediments in the transplantation of iPSCs. The transplantation of autologous iPSC-derived grafts or HLA homozygous iPSC-derived grafts has been the only option. However, it is impossible to generate these cell lines for broad clinical use due to different engraftment rates, long preparation periods, and high costs. Therefore, it is essential to generate universal iPSC-derived tissues regardless of the HLA haplotypes. A very promising study was recently published using a novel CRISPR/Cas system to generate HLA-edited iPSCs [48], resulting in HLA-C-retained iPSCs, which could circumvent attacks from T-cell and NK cells. According to the authors, these cell lines are immunologically compatible with >90% of the population, rapidly enhancing their therapeutic use.

Last but not least, the generation of suitable 3D microenvironments for proper chondrogenic differentiation is equally essential. The latest studies offer innovative strategies including cell cultivation in 3D ultra-purified alginate gel or bioactive biomaterials. Moreover, 3D bioprinting could serve as a valuable player in the field of scaffold composition supporting chondrocyte growth [49]. 

However, it is very challenging to generate functionally and structurally compatible AC tissue with the ability to bear full joint movement and biomechanical loading. Most studies rely on cost-effective rodent models, whose physiological properties differ significantly from humans due to the small join size and thin cartilage. On the other hand, experiments on large animals including horses, sheep, dogs, and porcine models benefit from a more substantial joint composition. Still, their use is often accompanied by high costs and difficulties in animal handling [50]. Each animal model has its advantages and limitations; therefore, it is essential to evaluate its properties prior to preclinical trials. To accomplish this goal, cooperation in various fields of expertise such as cell biology, tissue engineering, and clinical, is inevitable.

## Figures and Tables

**Table 1:** Comparison of different types of stem cells.

Cell Type	Pros	Cons
Autologous chondrocytes	Immunocompatibility	Limited availability
	Patient specificity	Low proliferation rate
	Native phenotype	Donor site morbidity
		Invasive cell harvesting
		Fast phenotype changes in vitro
MSCs	Large availability	Heterogeneity
	Various tissue sources	Reduced proliferation capacity
	No ethical issues	Differentiation difficulties
	Non-invasive cell harvesting	Age-dependent
	Additional paracrine signaling potential	Less effective cartilage regeneration
iPSCs	Unlimited cell source	Possible tumorigenicity
	Pluripotency	Lower differentiation efficacy
	Patient specificity	Genome instability
	Reduce immune response	Difficulties in obtaining uniform mature cell population
	Non-invasive harvesting of donor somatic cells	
	High throughput generation	
	No ethical issues	

**Table 2:** Overview of different study design applying iPSCs in osteoarthritis (OA) research.

Starting Cell Type	Differentiation Technique	Duration of Differentiation Technique	Costs	In Vivo Model	Results	Ref.
Chondrocytes from OA patients	EB formation from iPSCs and subsequent co-cultivation of TGF-1β transfected iPSCs with chondrocytes in alginate matrix	33 days	Less expensive	Subcutaneous transplantation of iPSC-chondrocytes into the dorsal region of BALB/c nude mice	Improved chondrogenesis; in vivo cartilage tissue formation	[35]
Human dermal fibroblasts	EB formation from iPSCs in 3D pellet culture	42 days	Less expensive	-	Alcian-blue and toluidine-blue positivity of differentiated pellets; on day 28, internal necrosis	[31]
ASCs; fibroblasts	Direct induction of iPSCs into iPSC-MSCs + chondrogenic diff. medium	49 days	More expensive	iPSC-derived chondrocyte transplantation into the osteochondral defect of the distal femur of nude athymic rats	Remodeling of the defect; active production of the hyaline cartilage matrix	[32]
Integration-free hiPSC lines 409B2 and 604B1	iPSCs cultivation in scaffoldless suspension culture supplemented with BMP2, TGF-β1, and GDF5	42 days	More expensive	Subcutaneous transplantation of iPSC-derived cartilaginous particles into SCID mice and joint defects of immunodeficient rats and mini-pigs	Pure cartilage tissue formation in vivo with high intensity to safranin O staining and positive collagen type II expression	[20]
Human neonatal fibroblasts	iPSCs cultivation in chondrogenic diff. medium supplemented by activin-A, WNT3A, Follistatin, BMP4, GDF5, FGF2, and neurotrophin 4	21 days	More expensive	iPSC-derived chondrocytes transplantation into cartilage defect of NOD/SCID mice	In vivo hyaline cartilage formation and regeneration; large tumor formation	[36]
Dermal fibroblasts from OA patients	EB formation from iPSCs	21 days	Less expensive	iPSC-derived chondrocyte transplantation into MIA-induced OA rat model	Increased production of aggrecan and collagen by grafted cells; improvement of movement abilities; persistent dyskinesia	[19]
Printed iPSCs	3D bioprinted iPSCs with co-printed irradiated human chondrocytes using a nanofibrillated cellulose + alginate (NFC/A) composite bioink, growth factors-GDF5 + BMP2 + TGFβ1	35 days	More expensive	-	Hyaline-like cartilaginous tissue formation	[37]
Mouse iPS cell line	3D cultivation in ultra-purified alginate gel + iPSC- derived MSCs; chondrogenic diff. medium supplemented by BMP-2	28 days	More expensive	Subcutaneous transplantation of iPSC-MSCs into the dorsal flank of nude mouse models	Positivity of transplanted grafts for Alcian blue; low positivity for type II collagen	[38]
Human iPSC lines	Direct chondrogenic induction of iPSCs by L2C4S4 bioceramics	14 days	More expensive	-	Significant reduction of differentiation time (14 days); prevention of hypertrophy of iPSC- derived chondrocytes; expression of Col II, Aggrecan, SOX9, and Col X/MMP13	[39]
